# Use of Quinine in Dropsy

**Published:** 1842-01

**Authors:** 


M. Levy, of the Military Hospital, Val de Grace, Paris, commends very highly the use of quinine in the dropsy which follows intermittent fever, and which generally depends on enlarged spleen. M. Levy believes that the spleen is often engorged before the first paroxysm of fever, and he says that close enquiry will generally satisfy us that there is pain and tenderness in the left hypochondriac region, and that the enlarged spleen can frequently be felt or detected by means of percussion. Whenever this is the case, when there is pain, tenderness, or manifest enlargement of the spleen, he advises cupping to the part, repeated from time to time, till these symptoms disappear. Then he gives the quinine. If dropsy supervene after the fever is broken, whether it be ascites, anasarca, or both, he gives the quinine in moderate doses and perseveringly. This will generally remove the engorgement and cause the absorption of the fluid. Mr. L. gives two cases to illustrate these views. His opinions were new to us, though published some time ago. We commend them to the notice of our country readers, under whose care many cases of intermittent will come at this season. In particular would we advise the early and careful examination of the region of the spleen, both by touch and by percussion. This, by the way, is one of the cases in which the auscultory percussion, as first practised by our friends, Drs. Clark and Camman, of this city, and of which a notice appeared in the 4th No. of the N. Y. Journal of Medicine and Surgery, would be useful. The spleen being carefully examined, if there is any pain, tenderness, or enlargement, let the part be freely and frequently cupped till the symptoms are relieved. Then quinine will be proper.

As to the use of quinine in dropsy, it is contrary to established rule, and if tried, it should always be done cautiously. A cautious trial, the authority of M. Levy, whose experience as a Military Surgeon qualifies him to speak pretty confidently, would justify.—*New York Medical Gazette.*

